# Precision justice: An imaginary of data and justice

**DOI:** 10.1177/03063127251342275

**Published:** 2025-06-14

**Authors:** Margarita Boenig-Liptsin

**Affiliations:** Department of Humanities, Social and Political Sciences, ETH Zürich, Zurich, Switzerland

**Keywords:** data justice, co-production, algorithmic decision-making, distributive justice, sociotechnical imaginaries, risk, information society

## Abstract

In Aldous Huxley’s 1932 novel *Brave New World* every person is conditioned through technologies to fit their social role, which is the source of the society’s alleged stability and rightness. This utopia of ‘precision justice’ is alive today in projects that deploy data and algorithmic models in decision-making in diverse branches of life to realize computing’s promise to create more just societies. I identify the sociotechnical imaginary of ‘precision justice’ by analyzing the promise and contestation of the use of an algorithmic model to calculate exam grades in the United Kingdom during the Covid-19 pandemic. ‘Precision justice’ is the product of the coupling of a normative concept of just distribution with data practices of identification and risk assessment, and is characterized by interventionist action, optimal distribution, and system management. It crystallized in the contexts of the emerging ‘information society’ in the 1970s United States, when visions of the risks and opportunities of information in digital form converged with the popular theory and practices of distributive justice. At stake in this imaginary is the model of the human with which it operates and that it reproduces. Instead of keying people to a substantive and expansive concept of justice, the union of distributive justice and data practices bind people to indicators and allocate them to specific places in society. To move beyond precision justice, this article calls for the need to look at justice and data symmetrically, as a simultaneously epistemic and normative set of concerns that must be addressed together in terms of what worlds we want to build.

During the Covid-19 pandemic in the United Kingdom, as public health measures prevented students from sitting for the university entrance exams, the government faced a dilemma: How would students receive grades that determine whether they can attend university? The UK government decided to assign students a ‘calculated grade’ created using an algorithmic model developed by the Department of Education and the Office of Qualifications and Examinations Regulation (Ofqual), the UK agency responsible for maintaining qualifications standards. The model calculated the grade on the basis of each student’s previous performance as assessed by their teachers, and standardized these assessments with the aim to curb grade inflation (Ofqual, 2020a). When the grades were announced, many students, families, political leaders and educators voiced concern about the results and the use of an algorithm to compute the grades. Students and their supporters came out to the streets in protest, some carrying signs with slogans that read ‘Your algorithm doesn’t know me’ (Chattle, 2020).

This is one of many examples discussed in scientific and popular media of perceived discriminatory outcomes resulting from reliance on inappropriately biased data or algorithmic models. The slogan ‘Your algorithm doesn’t know me’, however, calls attention to a dynamic that goes beyond the concern with discriminatory outcomes. With it, protesters suggest that the issue is not only *allocative* (about what you do or do not get) but *representational*, concerning how data stands in for the person (Barocas et al., 2017). At stake are judgments of who can use data, and how they can use it, to make knowledge claims about a person and what constitutes a legitimate representation of a person in data. By bringing into question the knowledge claims made through the algorithmic model, protesters contested a widely held sense that the algorithms serve justice.

These concerns were not evident when the UK government originally proposed ‘calculated grades’ in response to the canceled exams. Initially, the approach to use algorithms to decide the exam grade received wide support. Roger Taylor, head of Ofqual at the time, reflected that there was a ‘broad consensus in advance that it was the right thing to do’ (R. Taylor, 2021, p. 3). It was supported across party lines (Labour in Wales, Scottish National Party in Scotland, Conservatives in England, and the Northern Ireland Administration), by teachers’ leaders, universities, schools and colleges, and ‘even students, in advance of the results, could understand why it seemed the sensible thing to do’.

This broad consensus was understandable, as it has become standard practice for governments around the world to deploy data and algorithms for decision-making. Working alongside human operators, data and algorithmic models support innumerable decisions concerning the allocation of scarce resources, including vaccines, employment opportunities, or expert attention. This practice is bolstered not only by the availability of high volumes of data and access to computational power, but, importantly, by the *promise* that data and algorithms can and ought to benefit society.

Designers of the algorithmic models and authorities that choose to integrate them see multiple promises in computational tools, such as the promise to enable the efficient allocation of scarce resources or the promise to support human operators to make decisions in circumstances of apparent information overload. Algorithms are said to contribute to justice by introducing objectivity and transparency in decision-making processes to overcome human error, bias, or corruption (Mullainathan, 2019; L. Taylor, 2017) or make explicit the tradeoffs of values at stake (Kleinberg et al., 2019). Furthermore, algorithms offer the promise of ‘personalized’ interventions targeted to the specific situation and needs of the person in question. These linked promises of efficiency, manageability, insight-at-scale, objectivity, and personalization are the basis for claims that data and algorithmic models help to not only solve problems through the knowledge they assemble, like calculating a grade, but to promote justice. But what *kind* of justice is being served?

This article examines the relationship between the knowledge that data and algorithms are designed to produce and the promises of justice that this knowledge is supposed to serve. I use as a touchstone the promises and contestations of data and algorithms in the controversy about calculated exam grades in the UK; I refer to them as the ‘A-levels case’, because of the prominent A-levels university entrance exam. Importantly, this case unfolds during the emergency of the Covid-19 pandemic. The pandemic created new needs and opportunities for governments to experiment with data and computational approaches to matters of public life (Hantrais et al., 2021). Citizens in the pandemic questioned the legitimacy of their governments and scientific and technical experts, and sometimes specifically raised issues of trust, legitimacy, and expertise in relation to algorithms in decision-making. During the pandemic, injustices related to science and technology like vaccines, public health measures, or algorithms became national and global areas of inquiry and contestation by publics, political leaders as well as academics. Scholarship on data, algorithms and justice (henceforth ‘data and justice’) has provided repertoires for publics and governments to make sense of their decisions and experiences. This article is in conversation with some of these approaches to data and justice.

The sense of data and justice operationalized and contested during the Covid-19 pandemic is a result of a decades-long development of specific political commitments pursued through data analysis and computation. The epistemic ideal of algorithmically optimal rightness comes together with a political conception of rightness to produce a specific and prevalent sense of justice in contemporary Anglo-American societies. I call this sense ‘precision justice’ and I situate it in a longer social history of computing and political theory since the 1970s. ‘Precision justice’ is a utopian vision characterized by interventionist action, optimal distribution, and system management that links the promises of 20th century ‘information society’ with that century’s ideas of distributive justice. It crystallized in the contexts of the emerging information society in the 1970s United States, coupling data and computing practices of identification and risk assessment to a popular normative theory of just distribution. I trace how precision justice emerges as a variant of the same Anglo-American rationality shared by the technicians and analysts of the early information society and theorists of justice like John Rawls.

As we follow the promised logic of precision justice across cases—better data leads to better identification leads to just outcomes—we arrive at a situation where every person is so well understood in their unique preferences, qualities, and dispositions that it is possible to provide them just the right amount of just the right remedy or resource at just the right time. The term ‘precision justice’ evokes ‘precision medicine’, which the U.S. Food & Drug Administration (2018) defines as ‘an innovative approach to tailoring disease prevention and treatment that takes into account differences in people’s genes, environments, and lifestyles’ and has the goal ‘to target the right treatments to the right patients at the right time’. ‘Precision justice’ involves a similar aim to align the right intervention with the subject in time by identifying individual differences. The telos is a specific kind of perfect justice and perfect society—both *perfectible* through increasingly precise identification and targeted intervention. Thus, I also use the term ‘precision justice’ to signal frugality. Precision justice is an efficient and calculated justice that is premised on the need to precisely identify what each person is owed and what they owe—no more and no less.

At stake in precision justice is the model of the human that it operates with and reproduces. Instead of keying people to a substantive and expansive concept of justice, the union of distributive justice and data practices bind people to indicators and allocate them to specific places in society. Protests that challenge regimes of data and algorithmic representations today, such as in response to the calculated grades in the UK, identify what is problematic about precision justice for the human subject. Such protests are urgent calls for scholars to look at justice and data symmetrically, as a simultaneously epistemic and normative set of concerns that must be addressed together. I conclude with a call to think about and act in digital societies towards justice—and the human—beyond precision.

## Data and justice in the pandemic

In 2020, societies around the world experienced a forced experiment in digital life, as a result of the Covid-19 pandemic. At the same time as the deployment of calculation in the social world was expanding, its limits were thrust into view. The move to virtual sociality across all domains of life during the pandemic exposed and exacerbated the differential valuation of human life in societies: between rich and the poor, women and men in the labor force, educated elites and the marginalized, residents and migrants, and majority populations versus ethnic and racial minorities. Even over the short period of time since the beginning of the pandemic, wealth has become more concentrated in fewer hands. And the forces of authoritarianism, supported by tsunamis of disinformation on social media, have gained strength, while trust in many established institutions have been eroded.

In and around this troubled context, new calls for justice have echoed those of the US Civil Rights movement and the European student uprisings of the 1960s. Activists in the street and scholars studying how the promise of data for justice can go wrong have contested many assumptions about algorithmic promise. Speaking at a conference on responsible computing amid the pandemic, Williams (2020) described how societies have come to function based mostly on what can be calculated while ‘filtering out’ what cannot. People may be ready to admit that there may be something beyond the calculable, but it is said to be irrelevant for action. She described the phenomenon, and the consequences of the loss: ‘What is ruled as excess … is lost. Our technology cannot read this. It’s the excess that must be brought to a child custody hearing or to care for a Covid patient’ (Williams, 2020). In the context of the pandemic, scholars and activists highlighted the need of care and dignity and drew a sharp distinction between these basic necessities and the perceived coldness and efficiency of bureaucratic systems aided by algorithmic decision-making.

In recent years, three scholarly approaches—at times synergistic and at times conflicting—have been prevalent in how publics and scholars made sense of data, algorithms and justice issues. One approach focuses on identifying and correcting bias in algorithmic models. Another approach describes how data and algorithms disrupt allegedly normal processes of justice. And a third approach draws attention to colonialist and capitalist social structures built over decades, preparing the conditions for the latest data-related injustices. Together, these address how data collection, visualization, and analysis have been entangled in struggles for racial and social justice because they can make injustice visible, and thus actionable.

All three approaches have named important problems in the relationship between data and justice—‘bias’, ‘disrupted justice’, ‘structural injustice’—and pointed to ways to address them. Computer scientists, public administrators, and laypeople have mobilized these approaches in efforts to recognize and counteract the effects of oppression. I want to position the contribution of this study, to account for the mutual configuration between epistemic-technological and normative-political conceptions of justice, by discussing these three approaches and how actors in the A-levels case picked them up as registers with which to interpret their situation.

In the late 2010s, studies of the justice of decisions informed by data and algorithmic models attracted widespread attention, mobilizing public debate and protest (Amoore, 2020a; Eubanks, 2018; Noble, 2018). Prominent work in data and justice like Angwin et al.’s (2016) analysis of the COMPAS recidivism score algorithm or Obermeyer et al.’s (2019) analysis of a risk assessment algorithm in healthcare focused on identifying the allocative consequences of the models and their disparate impact on different social groups, frequently referred to as ‘bias’. Having identified the biases of the algorithmic model, an important strand of work in data and justice sets about trying to remove them through adjustments in the model or in the training and input data (Kleinberg et al., 2016; Mitchell et al., 2021; Stoyanovich et al., 2016).

The bias approach to issues of data and justice aims to correct the inputs into the algorithmic system to improve its outcomes. It has been criticized for the way that it does not take issue with the system overall (Barocas et al., 2017). By focusing on allocative consequences and disparate impact, the bias approach to issues of data and justice perpetuates a view that decision-making is only informed by data, instead of considering how the represented decision-making process forms or pre-configures the very possibility of decision and its aims through the kind of knowledge that it produces.

In the A-levels case, many analysts accused the algorithmic model used in the moderation of the grade to have produced biased results, creating a systematic disadvantage for students from larger state schools and bestowing further advantages to students from elite private schools (Jones & Safak, 2020). Ofqual research found that of the 39% of students whose grades were downgraded by the model compared to their teachers’ assessments, poorer students and those from marginalized backgrounds and from state schools were more likely to be affected (Adams et al., 2020). But these findings of bias have been disputed. Ofqual claimed that it had tested the algorithmic model on the specific definition of fairness mandated by the government. A report by the UK Office of Statistical Regulation that looked retrospectively at the 2020 A-levels controversy found that:

All the regulators carried out a variety of equality impact analyses on the calculated grades for potentially disadvantaged categories of students at an aggregate level. These analyses were based on the premise that attainment gaps should not widen, and their analyses showed that gaps did not in fact widen. Despite this analytical assurance, there was a perception when results were released that students in lower socio-economic groups were disadvantaged by the way grades were awarded. In our view, this perception was a key cause of the public dissatisfaction. (Office for Statistics Regulation, 2021)

This statement shows that irrespective of what constitutes a problematic form of bias (or, which definition of fairness Ofqual should aim to satisfy), the issue of bias played a significant role in the public perception of injustice in this case. Concern with bias in the data and justice relationship and work to address it at the level of data, algorithmic model, and definitions of fairness were a powerful repertoire behind the protesters’ claims that ‘Your algorithm doesn’t know me.’

Recognizing both the power and limitations of attention to bias in matters of data and justice, the ‘disrupted justice’ approach sought to step outside of the system’s frame to assess changes to commitments to justice in the digital age. For example, Dencik et al. (2022) examine how datafication has created what they see as a ‘disruption’ of what justice claims are, who can bring them, and how they can be addressed. They inquire into how, ‘as activities and behaviours are turned into data-points used to assess, evaluate and process outcomes, the terms upon which we not only access basic needs, but come to reason about value, attribute and receive recognition, and participate in processes that govern our lives are put into question’ (Dencik et al., 2022, p. 128). To understand how data configures deep-seated aspects of collective life, specifically how the ontology (‘what’), scope (‘who’), and procedure (‘how’) of justice shifts with datafication, Dencik et al. deploy Fraser’s (2008) concept of ‘abnormal justice’, a situation in which the first order conditions of justice are themselves in dispute. They argue that datafication represents a ‘dislocation’ and ‘disruption’ to each of these core components of what Fraser calls the ‘grammar of justice’: what value means and how people reason about it, who is the subject of justice and can make justice claims, and according to what criteria or procedures such issues can be settled (Dencik et al., 2022).

Claims about disrupted justice appeared prominently in student testimonies about their experience in the A-levels case. Students whose calculated grade was lower than the grade assigned to them by their teachers voiced frustration and deep uncertainty about the results and the algorithmic process used to arrive at them (Adams et al., 2020). As one student remarked, ‘I went and found what were the right courses for me, put my heart and soul into them, and to come out and be told, in this subject that you’ve been on track to get As and A*s in you’ve [*sic*] now got a C in, it’s just so frustrating.’ The student identifies an injustice in the rupture with the norm concerning what a just assessment and process for arriving at it would be. The sense of disrupted justice is due both to the algorithm and the pandemic, which prevented students from being able to sit for exams and earn their grade in-person. The dual conditions of algorithm and pandemic disrupted the standard process of this important passage from school to university for students and was a major factor in the ensuing protests.

While Dencik et al., following Fraser, draw attention to the unsettled space between some normal sense of justice before and abnormal state with datafication, another prominent approach, attending to ‘structural injustice’, emphasizes instead the continuities. The structural injustice approach points to the fact that data is less of a disruptor—going against the norm or proposing new norms—and more of a reproducer of norms present and latent in societies that retain racist or colonial legacies in the digital age (Benjamin, 2019b; Couldry & Mejias, 2019).

Benjamin’s (2019a) commentary in *Science*, on a widely read article by Obermeyer et al. (2019) that identified racial bias in an algorithmic model used to predict healthcare outcomes in the US, is an exemplary case explicating the structural injustice approach. Instead of developing Obermeyer et al.’s issue of bias, Benjamin focused her critique on the role of algorithmic optimization. She identified optimization as a defining feature and technique of applying algorithms to decision-making, which configures how justice is thought and enacted with algorithms, and called out a contradiction in what is being maximized and minimized by the model in question. Benjamin observed that while the health system should aim to increase health in the population, this aim is made actionable by the model that predicts a label of health cost, which must be minimized.

This contradiction makes sense in an institutional context that focuses the category of risk on the individual, which is a structural condition of risk and insurance systems in the United States. By analyzing the relationship of data and justice within situated historical and institutional contexts, Benjamin reveals powerful continuities across social and technological eras that are built into those institutions over time. From this vantage point, to counter the racial injustice of the algorithmic risk assessment system requires re-thinking the very category of risk. ‘It is vital’, she writes, ‘to develop tools that move from assessing individual risk to evaluating the production of risk by institutions so that, ultimately, the public can hold them accountable for harmful outcomes’ (p. 422). By taking the construction of risk seriously, Benjamin goes beyond Obermeyer et al.’s claims of racial bias in the ‘mechanism’ of the algorithmic model, explaining the broader ways in which the functioning of the risk communication system perversely aims to minimize health costs to the system to allegedly achieve better health for a patient. Her analysis draws attention to what—and who—is valued and what and who pose a risk to that value, and how algorithmic models come to adjudicate the value(s) calculus—numerical *and* moral.

Many analysts of the A-levels case drew attention to structural injustice as the root and feature of the debacle. For example, Charlotte Alldritt, director of the Centre for Progressive Policy, said: ‘The events of 2020 plunged the government into a trap of its own making. The system delivered to replace exam results merely replicated past patterns of inequality and attainment gaps between advantaged and disadvantaged children across the country, showing a blatant disregard for individuals’ (Adams, 2021). Alldritt alleged that the model picked up and perpetuated the patterns of advantage and disadvantage in UK society formed along racial, immigration, and class lines. For her and other observers, structural injustice not only reproduced inequality but also prevented the grading process from being able to recognize the effort and specific situation of the individual student. The structural injustice critique, however, is not aimed specifically at using algorithms in assessments, but equally to problems with grades by teachers. Researchers at University College London and the London School of Economics found that among their cohorts of students, those whose parents had graduated from the two universities received on average 15% higher teacher-assessed grade than through the Ofqual algorithmic model, showing how structural advantages accrue to those who are already in the elite education system (Anders et al., 2021). As for Benjamin, the structural injustice critique identifies the problematic power of representations like risk or grades beyond the fairness of any one measure, and advocates a deemphasis on the relative value of these representations in public institutions.

The ‘bias’, ‘disrupted justice’, and ‘structural injustice’ critiques of data and algorithms in decision-making contexts each provide a reading of the problematic relationship between data and justice in the statement ‘Your algorithm doesn’t know me’ in the A-levels case. By aiming to explain how data and computing over time had participated in the production of the very order deemed just, I provide a contrasting articulation of the injustice expressed in the protesters’ statement: The algorithmic model is not bad because the data is unrepresentative (the biased data approach), nor because the model is decoupled from the regimes of verification and legitimacy of pre-digital institutions (the disrupted justice approach), nor because the data and algorithmic model derive from historic patterns of domination (the structural injustice approach). Rather, protesters appear to resist the very terms of moral and epistemic rightness encoded and enacted in the promised knowledge with the algorithm.

## The co-production of data and justice

The UK protesters’ statement about calculated grades, ‘Your algorithm doesn’t know me’, expresses concern with a configuration of data and justice that has been developed over decades. It represents a continuous and structural feature of how justice is put into action with data and algorithmic knowledge. Here I set out to identify and characterize the sense of justice in algorithmic promises. I am interested in how an idea of justice is co-produced (Jasanoff, 2004) with data and algorithmic models and through the work they do in social institutions. The co-productionist perspective supports inquiry into how a certain kind of justice is enacted through the data projects themselves, even if they appear to not be ‘explicitly connected’ (L. Taylor, 2017) to a predefined idea of justice as held by the agencies and authorities that enact them. By attending to how different actors make claims about the justice of algorithmic practices and outcomes and how people challenge these claims in public, we can see how data and justice are mutually configured. I trace the linked epistemic and normative contours of the *promised* paradigm of data and justice in the longer-term trajectory of how social actors have questioned and responded to problems of justice in contexts of information societies.

The coupling of data and justice in social practice reflects and reproduces a ‘sociotechnical imaginary’, where sociotechnical imaginaries are ‘collectively held, institutionally stabilized, and publicly performed visions of desirable futures, animated by shared understandings of forms of social life and social order attainable through, and supportive of, advances in science and technology’ (Jasanoff, 2015, p. 4). In my case, I am particularly interested in visions of justice and of just societies within broader visions of desirable futures, and how these are co-produced with data and algorithmic models for decision-making. The framework of sociotechnical imaginaries builds upon co-production and attunes analysts of technology and society to how normative and epistemic commitments are achieved together in and through social imagination. Sociotechnical imaginaries function as unifying intersubjective forces that condition the forms of life on offer in a society and the directions in which a society develops.

Imaginaries gain animating power in human affairs by remixing discursive, material, practical, and institutional elements in the collective and intersubjective space of promise and imagination, producing new representations whose circulation and work needs to be studied. From an STS point of view, remedying whatever injustices are built into the system requires uncovering the implicit imaginaries and alliances of data and justice and pointing to how they come to be stabilized, sustained, and strengthened. That work can then support political conversations in communities to determine how to reconnect and realign collectively held visions of just societies and data practices.

## Data will make it right, just in time

Public concerns about social justice and the promises of data and computing for society came together in the controversy surrounding the UK government’s decision to replace the annual university entrance exam with a ‘calculated grade’ for all graduating students. On March 18, 2020 the UK government announced that the exams would be canceled (Walker & Adams, 2020). It held a series of consultations and formed a consensus that a calculated grade would be delivered. The chief concern of the government was grade inflation, because of teachers’ incentives to report higher grades for their students to help their chances to secure a university place (Ofqual, 2020a; Anonymous, 2020). In some schools, the information about how many students attend which universities is used to assess the school’s and teachers’ performance, creating an incentive for teachers to give higher grades. Related to the issue of grade inflation, the government administrators were also concerned to stay within reasonable university enrollment numbers (Adams, 2020). Government authorities had recently told university administrators to cap their enrollment (Adams, 2020; Department of Education, 2020), so grade inflation would make it challenging for universities to fulfill those caps. The UK government produced ‘calculated grades’ for all students and announced them on the annual ‘grades day’ on August 13, 2020 (Ofqual, 2020b). There was an immediate public outcry that the grades were unjust. After a few contradictory back-and-forth responses regarding policies of how students could appeal the grades, on August 17, 2020 the government turned around and announced that it would award students the higher of their teachers’ assessed grade or the calculated grade (Ofqual, 2020c).

This sequence of events to produce the ‘calculated grade’ is a real-world example of the fits and starts of the use of algorithms in a decision-making process. It is marked by three technical and procedural features: interventionist action, optimal distribution, and system management. These three features make up the public representation of the process of algorithmic decision-making that both emerges from and reinforces the precision justice imaginary. As I show below, each technical feature also embodies and reproduces a form of normative rightness.

One feature of the promise of algorithmic decision-making that we can see from this case is that data and algorithmic models are intended to support targeted and timely *interventions* into sociotechnical systems. An intervention is a specific type of action: one that comes in from the outside in the course of things. In an intervention, a person enters activity already underway to alter its course as a corrective or reparative action. In the A-levels case, if the grades, as a core input into the system of university entrance management were missing because exams had been canceled, then the algorithmic model could be used to plug into that system and produce the ‘calculated grade’ that could be used instead.

Whether the intervention is to correct the flow of gas through a pipe or shape a person’s life path by providing them with a health check or counseling service, it occurs in a discrete moment in time for which it is precisely calculated. As Cohen argues in her account of the development of public numeracy, the idea that human beings can successfully intervene in nature is linked to quantification as a tool for making the intervention and assessing its results (Cohen, 1999, p. 108). Importantly, a calculated intervention is not a wholesale reformation or revolution, not a full reconfiguration of a system or a life. As analysts remarked in the A-levels case, the government did not seriously consider making changes to the system overall, such as canceling grades for that year or increasing the number of seats in universities (R. Taylor, 2021). In other words, an intervention is consistent with the task of redistribution of resources, which does not require one to consider the nature of the resources or change their overall quality or amount. Rather, it is a tactical, targeted adjustment directly at the site of need in a time of need, whose risks and benefits are calculated as precisely as possible. Like the justice that it dispenses, the interventionist action is frugal and seeks to preserve the status quo as much as possible.

Second, we can see in the A-levels case how the action of intervention goes hand in hand with the aim of *optimization* that guides algorithmic decision-making. The intervention determined by an algorithmic model is an optimum: the result of a minimized loss function (or, inversely, maximized utility or fitness function) that represents the cost (or, inversely, utility) of an event. In the A-levels case, the UK government used the model to precisely optimize the allocation of university seats to students. The decision-making problem that the model was brought in to solve was framed as a scarcity problem: a limited number of university seats. University administrators were told to stay within the capped number of seats (Anonymous, 2020) and the algorithmic model helped to support this by standardizing the teacher-given grades to bring them within the necessary limits. Each grade, in this sense, served as an optimum: a balance between what the student deserved based on their previous work as reflected in the teacher’s assessment, their rank relative to other students, and all adjusted to the number of available university seats. In promissory accounts of algorithmic decision-making across sectors, the algorithmic optimum is equivalent to what is deemed the socially optimal action, forming the union of epistemic and normative rightness in precision justice.

The task to design a model that would find the optimal grade for each student in the context of existing data and constraints draws attention to a third feature of the promissory representations of algorithmic decision-making. This is the *systems view*, or the idea that intervention and optimization are the linked pair of action and aim that are put to work in a broader context of education (or, alternatively, health care or criminal justice) conceived of as a ‘system’ that requires ‘coordination’ and ‘management’. The system is characterized by a constant flow of data that represent the fragmented traces of people’s characteristics and behaviors. Intervention and optimization serve to focus attention on the system’s proper functioning, and in doing so they take away attention from inquiring to what ends and for whom the system is working.

This was prominently on display in the A-levels case, when the UK government developed the algorithmic intervention based on its own understanding of what exams are for rather than considering the point of view of the individual citizen (R. Taylor, 2021). ‘For policy makers and administrators,’ Taylor wrote, ‘the question was, how do we find a way of enabling young people to progress? From this angle, the question quickly becomes how to fill the information gap left by exams, what information can be used instead, how to make sure the system doesn’t break down’ (p. 6). On the one hand, the systems view can be expansive and comprehensive, taking into account the situations of millions of individuals and harmonizing them with the broader interests of institutions and processes. On the other hand, the nature of the comprehensive insight from a systems view can be myopic in how it privileges a point of view from which to see the situation and ignores how the situation is perceived from the point of view of others situated differently, such as the individual student. Seen from a student’s perspective, the exam was the opportunity to demonstrate that they deserve a university place—an opportunity to ‘produce the evidence they need to claim their place at university’ (p. 7). Taylor admits that the government did not adequately consider this citizen’s point of view, for which the calculated grade would not have been a solution. Instead, they opted for the formulation of the problem in terms of ‘How do we plug the gap in the machinery and work out which pupils go in which places?’ This systemic conception of the problem (‘plug the gap in the machinery’) went hand in hand with the calculated grades as the solution.

Together, interventionist action, optimal distribution, and system management enacts the promise of data and algorithmic knowledge in the making of just political actions to claim: ‘The algorithm knows you better than you know yourself.’ In decision-making contexts like education, health and criminal justice, the algorithmic model ‘targets’ students, patients or defendants for the receipt of specific resources. This identification comprises a key object of algorithmic systems (Bates, 2024). Algorithmic decision-making produces an indicator, like the grade, that connotes a kind of risk assessment score to assess the likelihood of an intervention’s success or failure. This indicator labels the individual and informs what kind of intervention they ‘deserve.’ The task of the manager and coordinator of the system—the person whom the algorithmic model is aiding—is to ensure, as we have seen, the smooth functioning of the system, rather than of the individuals within it. Thus, while interventions are targeted at specific individuals, it is the system that must be watched over and managed. Precision justice concerns itself with the smooth working of the system, rather than any given person who is the bearer of the body and spirit upon which the system-optimizing interventions are performed. The subject is known to the system and within the system. Yet, the same features that make the subject known to the system also subject people to targeted interventions based on results of optimization in ways that do not comport with the subjects’ sense of being adequately ‘known’.

The A-levels case is specific, but the features of the algorithmic decision-making that assemble interventionist action, optimization, and system management are regularly deployed in other settings. Consider, for example, accounts of how the use of algorithmic models can be beneficial in the areas of health and criminal justice. Algorithmic models are routinely used in health care, from diagnosing illness to identifying what kinds of therapies might benefit a patient to predicting who may fall ill in the future. One type of model in this last category of health services management is developed to help healthcare providers identify which patient may become critically ill and where an intervention before symptoms begin can help avert illness (Obermeyer et al., 2019; Secinaro et al., 2021; Shilo et al., 2020). When the algorithmic model works as it should, the promise is that it allows the healthcare system to identify the vulnerable individual and provide them with the targeted amount of medical attention to maintain the status quo of their health.

Practitioners justify algorithmic decision-making in the criminal justice system in a similar way. Models that predict a defendant’s recidivism rate, or the likelihood that a person will reoffend if released on bail, promise to help the system provide each defendant with the appropriate resources such as counseling and social services (Bennett Moses & Chan, 2018; Powell, 2018; Smith et al., 2017). Algorithmic risk assessment tools assemble diverse data about the individual and compare it against the behavior of aggregated records of individuals ‘like’ them. The stated aim is that this will yield a score that will help to identify the kind of intervention that will be most effective at preventing that individual from endangering the community. This data solution is predicated on precise identification of the person and the ability to compute their entitlements to the appropriate type of intervention, which would then correspond to a socially just outcome.

Algorithmic decision-making in practice varies significantly across areas of application, with distinct concerns and modes of implementation in domains such as health, criminal justice, economics, and logistics. Each context of application raises specific challenges around interpretation of the data, such as what a specific number or indicator means and how this information can be responsibly acted upon within institutions with specific mandates and range of possible actions. In light of this complexity and diversity, it is striking that there is a consistent schematic representation of algorithmic decision-making in promissory discourse and public imagination.

Across cases, the schematic representation of the algorithmic decision-making process is tinged with a normative promise. What is ‘good’ or ‘right’ is an intervention that is carefully calibrated to be proportionate (not more, not less) to the identified phenomenon according to the signals read from the data.

The same configuration of intervention, optimization and system that permits the claim ‘The algorithm knows you better than yourself’ is the one that is subject to vehement protest: ‘Your algorithm doesn’t know me.’ To understand the source of the authoritative power of the precision justice imaginary as well as the strength of conviction concerning its injustice, I turn to the historical epistemic and political dynamics that have helped to establish ‘precision justice’ and propel it to the present.

## ‘Precision justice’ in the making: Conceptualizing and enacting data’s promise of justice

What conceptual and institutional dynamics contributed to the present-day prevalence of the precision justice imaginary? I trace the development of the imaginary to 1970s American intellectuals and theorists like Daniel Bell and John Rawls, who advanced popular ideas about technology and justice in the context of the ‘information society’, used by other intellectuals to consider the promises and perils of the products of scientific and technological innovation.

‘Information society’ is a concept that sociologists, business leaders, and government officials used to designate an immanent new era of social, economic, and political relations defined by the production, circulation, and analysis of information among governments, industry, and publics (Bell, 1973; Masuda, 1981; Webster, 2006). They saw information as a resource and public good of complex economic and social value that promised to catalyze change in societies. To manage this important new resource and social transformation, scholars and practitioners resorted to approaches and practices of risk management and distributive justice, defining new technical mechanisms of computing architecture and data protection to enact the desired structures of access to information in digital form (Saltzer & Schroeder, 1975). This intertwining of promissory visions of information society with a culture of risk management and distributive justice paved the way for how justice and computing are linked in the imaginary of precision justice in the present.

While the excitement about computing for society shared much with the promissory discourse of science and technology across ages, the cautious stance of even the information society’s advocates reflected specific concerns about the contestations, catastrophes, and frictions with science and technology in their time. Starting in the 1970s, social analysts from different disciplines converged on the notion of risk as a common concern and defining concept for thinking about the age and the general sense of social precarity in it (Beck, 1986; Garland, 2003; Jasanoff, 1986). In this context, sciences and technologies, such as recombinant DNA, industrial pollution, pesticides, and nuclear technology, were understood as inherently risky enterprises, whose risks and benefits needed to be actively managed. Risk assessment (technical evaluation of risks) and risk management (decisions of what to do about them) became two linked techniques that experts used to justify the introduction of scientific and technological innovations into public life (Jasanoff, 1999).

To better understand and manage technology’s risks, in 1973 the US government established the Office of Technology Assessment (OTA). The OTA described technology as ‘increasingly extensive, pervasive, and critical in their impact, beneficial and adverse, on the natural and social environment’ (Technology Assessment Act, 1972, p. 797). It formalized the processes for evaluating technological impact and signaled a commitment to formal assessment that would inform Congressional legislative activity. In its early years the OTA took up many signature issues of science and technology including mass transit, nuclear proliferation, oil and gas production, and cancer testing. The first major assessment of computing and information technology came in 1978, with an inquiry into the topic of information technologies and criminal history (Office of Technology Assessment, 1978). While the OTA grappled with risks of technology in an expert and technical manner, some disasters demanded more public accounting about what happened and how it could be prevented in the future, and directly implicated ethics and justice.

One notable example from this period of deliberation on ethics and justice of science and technology was the Belmont Report. The Report was written by a commission tasked with understanding what went wrong in the US Public Health Service Syphilis Study at Tuskegee, conducted by the US Public Health Service from 1942 to 1972. The Report has had a lasting impact on how the risks and benefits of science and technology are perceived and how justice is considered in their light. It remains a reference point for discussions of ethics and justice of emerging science and technology (Adashi et al., 2018), especially for bioethics (Hurlbut, 2017) but also for data and computing (Metcalf et al., 2016; Paxton, 2020).

Important for how experts would frame questions of justice, science, and technology since, the Report established a link between scientific and technological risks, distribution, and justice. It named justice as one of the principles of ethics (alongside respect for persons and beneficence) that all scientists must adhere to when conducting scientific research. It focused on *distributive justice*: ‘Who ought to receive the benefits of research and bear its burdens?’ ask the report’s authors. They answer: ‘This is a question of justice, in the sense of ‘fairness in distribution’ or ‘what is deserved’’ (National Commission for the Protection of Human Subjects of Biomedical and Behavioral Research, 1979, p. 8). The Report posed the question of justice in terms of a ‘who’ and answered it by suggesting that individual people’s qualities (need, effort, contribution, merit) are of primary import for the just distribution of science’s burdens and benefits. Concern about the distribution of benefits and burdens of research also inform the principles for the respect for persons and of beneficence, the other two ethical principles named in the Report, underscoring the centrality of the distributive frame to protect human subjects from the risks of science and technology.

The Belmont Report’s formulation of justice in terms of distribution corresponded with the rise in the post-World War II United States of the theory of distributive justice (Fleischacker, 2004, p. 80), and especially after the uptake and circulation of Rawls’s (1971) *A Theory of Justice* by political and economic thinkers and practitioners. The growing recognition of societal differences and disagreements in the United States of the 1960s and ’70s, from racial and gender discrimination made publicly manifest by the Civil Rights Movement to issues of civil disobedience concerning the Vietnam War and positions on nuclear weapons, presented the need to solve the problem of distribution while recognizing individual differences. Distributive justice theorists espoused the view that societal inequities are the result of unfair distributions of resources (Forrester, 2019). Improving the fairness of distribution, something that Rawls’s theory, in particular his ‘difference’ principle, set out to do, aimed to fix the inequities and bring society into alignment. Distributive justice provided a way to judge such distributions, and, in turn, to use such judgments to judge the justice of social institutions responsible for re-distributing resources (Jackson, 2005, p. 359). Putting fair distribution in practice in the hands of economists and economic-minded political scientists called for and depended upon modes of calculation—especially the calculation of difference.

Practitioners concerned with justice took up information as a tool to aid in their distributive projects. The importance of information for distributive justice becomes particularly apparent when we consider the related notion of social equity. Social equity says that differences among individuals need to be considered to treat people fairly. It stands in contrast to equality, or the idea that all people of a certain equal standing (e.g. all citizens) need to be treated the same. The notion of social equity arose in the United States in the 1960s, as a concern for public administration and in the context of the Civil Rights movement’s demands for gender and racial justice (Gooden, 2014). Following American historian George Frederickson’s prominent suggestion in 1968 that the principle of equity be the ‘third pillar’ alongside economy and efficiency in the practice of public administration, researchers began to debate how to mobilize the concept (Gooden, 2015).

Considerations of equity motivated the need to assess individual differences. Economists concerned with income distribution observed that evaluating distributive equality is a ‘mechanical or statistical matter’, but that evaluating distributive *equity*, ‘implies the impossibility, in principle, of objectively judging whether something called maldistribution exists in a given society and whether one distribution is better than another’ (Bronfenbrenner, 1973, p. 10). For example, economist Joseph Stiglitz explored in a 1975 paper the advantages of identifying and providing information about individuals to more justly distribute wages in correspondence with each person’s ‘true marginal product’ (Stiglitz, 1975). To achieve equity of distribution, analysts require to know specific characteristics of people and how they compare. Statistics and computation could respond to this need for precise measurement and identification of qualities with individualized risk assessment. Though mainly justified by the accuracy or efficiency of their results (rather than appeals to their relative fairness or justice as compared to non-computer methods), the use of computers to identify risks for specific populations and individuals in medical or criminal justice contexts took off in the 1970s and ’80s (for examples of computing in medical diagnosis, see Barnett et al., 1987; Weiss et al., 1978; in criminal justice, see Dunworth, 2001). A focus on fair distribution to achieve justice, on the difference principle and on equity, requires and invites the tools of identification to describe the uneven lay of the world in such a way that it can be made level.

Pursuing justice in advanced technological societies through distribution required not only the equitable distribution of the risks, but also of the perceived *benefits—*the ‘goods’— of science and technology. In the 1970s, a new public good became the center of scholarly and political attention: computing. In his description of the ‘post-industrial’ society, sociologist Bell (1973) presented a world in which knowledge and information were the main organizing forces of social life and economic value. In the decades that followed, scholars of information, concerned about the equal distribution of this prominent ‘new’ resource, adopted Rawls’s theory of justice to account for information as a primary good (Hoffmann, 2017). If access to information was indispensable to the pursuit of one’s valued ends, then overcoming inequalities in the distribution of information was an imperative (Drahos, 1996). This imperative has been sustained since the 1970s by the normative and ontological ideas and ideals of information as a good, as flow to be channeled (Castells, 1996; Haraway, 1985) or to course ‘free’ unimpeded through the social body (Barlow, 1996), and its perceived role in society as the basis of a new economy and the means to individual flourishing and societal progress.

The confluence between justice in science and technology conceived in terms of distribution and information as that which is both the flux amenable to distributional forces and essential for operationalizing just distribution across social contexts continues today in the precision justice imaginary. In the contemporary imagination, data is brought to bear on problems by diagnosing a specific need and helping to regulate the flow of resources to set it right. The ingredients of the precision justice imaginary come in the twin mechanisms of the risk assessment system and distributive justice (as a means of spreading risk in a manner deemed fair), which, since the 1970s, are further activated through the technical possibilities and promises of information in society.

Having identified the features of the precision justice imaginary and the historical developments that have contributed to its formation, I now turn to its effects for people. What do the ways that student protesters and social theorists named and challenged these effects in the A-levels case further reveal about the imaginary?

## The human in justice

The promised logic of precision justice—the sense of justice that is both expected from data and algorithms and the subject of contestation in the A-levels case—is frugal. It is an efficient justice, calculated to apportion to each person according to what they owe and what they are owed. By getting right the balance of entitlements, data and algorithms can claim to ‘know you better than yourself’. With this claim, the sense of justice beyond precision is lost, leading some to retort: ‘Your algorithm doesn’t know me.’ When students shared their concerns of the 2020 A-levels with journalists and researchers, they repeatedly pointed to the problematic experience of being subjected to the algorithm’s gaze, not just the resulting grade. They challenged the very legitimacy of the algorithm to know them, or how the algorithm framed them as knowable subjects. By attending to the implicit model of the human in the precision justice imaginary, I show how the consequences of the imaginary are not only in the allocations it generates, but the assumptions it holds and effects it engenders on the *person* in digital societies.

Problematic effects of algorithmic models in decision-making processes have been extensively studied. Scholars have identified the tendency to efficiently *allocate a person* (in contrast to allocating resources to persons) to a specific place in society that is alleged to be right for them. For example, some studies have found pervasive ‘social sorting’ in everyday uses of data and computing tools by multiple agencies to understand and manage populations in diverse areas of application, from marketing to provision of social services to policing and education (Lyon, 2003). Others have meticulously documented the individual and societal harms that are produced by algorithmic categorizations (Benjamin, 2019b; Eubanks, 2018; Noble, 2018; Sweeney, 2013).

While expressing strong concern about social sorting and allocation of the person, scholarship on data and justice has not yet linked these effects to a specific model of the human implicit and operationalized in justice promised with algorithmic decision-making. To make this link, I first draw on Aldous Huxley’s science fiction and Jacques Ellul’s philosophy of technology. These thinkers’ warnings about the relationship of the person to justice, before discussions of an information society, articulate the problematic model that I wish to make visible. Second, I show how the conception of what is relevant to know about ‘the human’ in Rawls’s theory of justice is consistent with what is relevant (and possible) to know about ‘the human’ with information in digital form, especially for risk assessment purposes. The dynamics of information society and political theory of distributive justice contribute to a sociotechnical environment in which the model of the human subject as perfectibly knowable takes hold.

In his dystopian science fiction account of London in *Brave New World* (1932), where residents live satisfied, comfortable lives, Aldous Huxley presents a society where precision justice rules. This society is sustained by perfected application of chemical, media, and industrial management technologies. From the time that they were embryos, each member of this world has been chemically and psychologically conditioned for a specific niche. The result is that every adult takes pleasure in their work, which everyone else respects and finds to be a necessary contribution. Each person’s abilities match their roles, and this measured appropriateness is considered by most residents to be also a normative rightness of their society and source of its stability and prosperity.

Writing some twenty years after Huxley, Ellul (1954 [1964]) identified a similar dynamic in which the freedom of the person becomes subordinated to efficiency and order of the system. He observed that in societies where legal techniques are advanced and procedure-focused, the bureaucratic enactment of law takes over the incalculable, ambiguous, aspirational grounding of law in the pursuit of justice. Law and justice are separated, and law’s end (and spirit) moves towards securing order and efficiency.

For Ellul, the individual person is at stake in this transformation. In his account, justice results when the law enables each person to fulfill their function in society. When the law becomes a ‘mere organizer of individual functions’, however, the individual becomes subordinated to the functioning of the whole in a deterministic and unfree way (Ellul, 1954 [1964], p. 295). Ellul writes: ‘In affirming that there is no law without efficiency, we in fact announce the implicit sacrifice of justice and the human being to efficiency.’ Not only is justice sacrificed because of precision justice, but so is the human being. When the telos of justice reigns above the law, the human function is organized always in relation to it; without justice as the telos, the human herself becomes subordinate to efficiency. No wonder, then, that mothers and fathers do not exist in Huxley’s brave new London, that ‘everyone belongs to everyone else’, and that the special devices installed on the chimneys of its crematoria recapture the maximum amount of ninety-eight percent of the phosphorous from its dead, in order to extract the most from a human life: ‘Fine to think we can go on being socially useful even after we’re dead. Making plants grow’ (Huxley, 1932, p. 73). When brought to its tragic utopian end, a precision justice society is made up of atomistic, unfree, substitutable, and unmarked lives.

Huxley and Ellul’s texts not only serve as warnings about aspiring to justice and just societies with technology but identify specific consequences for the human in such endeavours. In the context of US political theory in the 1970s, with practices of risk assessment and the rise of information science, this perfectibly knowable model of the human became realizable in new ways.

In both the information science and Rawlsian frameworks, ‘the human’ is a combination of qualities that are common to all (and hence universal or generalizable for all people, or at least all members of a moral community) and qualities that are particular to the one. The particular qualities are conceived in such a way that they can be hidden from view, whether through the Rawls’s ‘veil of ignorance’ thought experiment or the technical practice of de-identification (in fulfillment of aspirations to justice or privacy) or brought into view through more precise identification and metrics (in fulfillment of values of equity or affirmative action).

Risk assessment functions with and performs a model of the human that treats each person as a discrete entity with theoretically all-knowable qualities. The ideal risk assessment indicator for a person in the context of any system (health, justice, marketing, education, etc.) is the score that best corresponds to their actual or predicted behavior. This would be a situation of zero statistical bias, implying a perfect (within parameters of error or noise that are deemed irrelevant for the uses of the score) representation of the person’s qualities. By collecting data about a person’s qualities and behaviors, using an effective statistical model to represent this data with an indicator, and deploying that indicator in the context of decision-making the analyst operationalizes this implicit model of the human with respect to the targeted person to produce the desired effect in the system.

Like the model of the human in risk assessment, Rawls’s theory of justice operates with a theoretical presupposition that the individual’s qualities are knowable and salient for just social distribution and organization. People in Rawls’s ‘original position’—the idealized state in which they arrive at the principles of justice, behind a ‘veil of ignorance’—know everything about how to reason about their interests in the world, even as they know nothing of the specific qualities and resources they have been allotted in life (Hurlbut, 2017). The model of the human in Rawls’s theory of distributive justice is both grounded in a presumed common environment and detached (or detachable) from it. This model of the human allows social actors to mobilize Rawls’s theory to derive shared principles of justice within a community (on the basis of the common) and the distribution of the resources to each person according to their allotment (on the basis of the particular).

Information, and data as information in digital form, is integrated seamlessly into the models of the human in risk assessment and in distributive justice. Risk assessment has benefitted from big data, as well as from developments in data analysis and algorithmic models, harnessing the power of computation to extend descriptive and predictive capacities—and its appeal as a decision-making tool. Information also fits neatly into both sides of the epistemic equation in Rawls’s original position. Information can be layered on the knowledge of the person in Rawls’s original position without upsetting or challenging the knowledge held in common. On the other hand, information as discrete markers that can be (de-)coupled at will from the object or person it describes (such as, for example, age, race, class, criminal history) can be operationalized through the veil of ignorance. Information about individual particulars can be either hidden from view when necessary to derive allegedly universal just principles or brought into play when, following those principles, individual considerations need to be brought into alignment with expectations about fairness. Rawls’s mechanism is not only a formula for arriving at principles of justice that are acceptable to the community. It is also a way of articulating what it is about the human that matters socially, for questions of how to organize societies through institutional decision-making aided by algorithms.

This model of the human is powerful because of how it can be operationalized. In contemporary cases of algorithmic decision-making, justice becomes a tweaking of the universal and particular layers and justifying why one variable was extended while another deemphasized (‘weighted’, in technical terms). In the A-levels case, the UK government developed the algorithmic model used to produce the grades from a variety of inputs and based on considerable expert work. The work involved precisely such careful adjustments to variables to realize the process and formula that would lead to the grades satisfying Ofqual’s mandated definition of fairness. Approaches to data and justice that work on bias mitigation frequently use such tweaking to remedy the misrepresentations of the algorithmic model (for bias correction in a model for recidivism risk scores see Corbett-Davies et al., 2016, 2017; Narayanan (2018); for a case of a health prediction model see Obermeyer et al., 2019). Work to correct biases and inequitable distributions they result in, however, does not challenge the normative regime that precision justice interventions are the right means to achieve good health or just verdicts, nor does it challenge the implicit model of the knowable human at the imaginary’s core. In efforts to correct biases, the precision justice imaginary persists, rather than being replaced by recognition of contingency, unknowability, and uncertainty as starting points from which to work out just approaches (Amoore, 2020b)—something that the students’ protest starkly demands.

The sociotechnical imaginary of precision justice is expressed as a dystopian fiction in the 1930s, is discussed in the 1960s as a worrying presence, becomes substantiated with new technical and normative mechanisms in the 1970s, and is extended and amplified with data and algorithms in decision-making across diverse social contexts since the 2010s. Unlike in Huxley’s novel, in the contemporary precision justice imaginary there is no single authority that determines a person’s place and, in many cases of algorithmic decision-making, no one is specifically interested to surveil, judge, categorize, profile, or act on the individual directly (Rouvroy & Berns, 2013). Rather this is the animating imaginary and outcome of the normal functioning of a system that reads all inclinations, choices, and ‘personalized’ needs as discoverable and actionable through data and computation. Beyond the disparate impact of *allocative* effects for an individual or community, the imaginary is significant for how, through its framing of the human subject, it lays down conditions of life in contemporary digital societies.

## Conclusion: Re-imagining the coupling of data and justice

In this article, I have asked what people are protesting when they say ‘Your algorithm doesn’t know me.’ What can their protest reveal about the promised way in which data and algorithms are imagined as leading to just decisions?

Protesters have challenged the regime of algorithmic knowledge, including the relationship between the epistemic promise of knowing accurately and the political promise of intervening justly. They have challenged the precision justice imaginary: ‘Precision justice’ is a collectively held, institutionally stabilized, publicly performed and technologically sustained vision of justice characterized by interventionist action, optimization, and systemic view. ‘Precision justice’ presumes and reproduces a view of the human subject as perfectibly knowable—a kind of knowledge of the person that becomes a precondition for enacting justice.

This imaginary is at work in contemporary digital societies, but it has a longer trajectory. It is a product of the meeting of 1970s US political theory, especially the theory of distributive justice, and the practices of risk assessment and the rise of information science. At stake in the imaginary is an idea of the human that it assumes and produces. In linking the expression of justice with the data-enabled increasingly precise identification of people in the imaginary of ‘precision justice’, we unwittingly strip away the recognition of the incalculable singularity of the individual and the bonds that bring together different people under that shared designation of ‘human being’.

Even as protests have challenged this imaginary and pointed to what Amoore (2020b) has termed ‘the future of political protest’ capable of responding to the ‘politics of the algorithm’, the role of data and algorithms in decision-making is on the rise. Following the proliferation of publicly available generative Artificial Intelligence (AI) tools, corporations and public administrations around the world are developing guidelines and best practices for the integration of AI into their institutions’ decision-making processes. Leaders tasked to create these processes are concerned with how to maintain the trust of citizens and consumers. In these circumstances, leaders recognize that what makes the use of AI in decision-making right, good, or just is specific to the workings of each institution. While it is essential to understand these specifics, this study has sought to also identify the broader continuities and mutual dependencies between epistemic affordances of data technologies and political commitments to justice that have co-evolved over time. Understanding how data and justice have come to be coupled in the course of recent history of information society and political theory of distributive justice in the United States, and how it has been expressed and challenged by intellectuals as well as publics in other sites, prepares the way for subsequent work to look for this imaginary in other contexts where data and algorithms are brought to bear on issues of decision-making.

If, as I have argued in this study, the relationship between data and justice is co-produced, then it needs to be addressed in terms of what worlds we want to build—without assuming that the ideals of justice articulated in the past are sufficient for dealing with the real and irrevocable shifts in our ways of representing the world in data. This study is therefore an invitation to ask about what alternative conceptions of justice look like and how data and algorithmic models might support them. Perhaps when, instead of focusing on achieving a more perfect justice through data, societies could subordinate data to projects of learning to embrace an imperfect humanity, we could move towards more just knowledge, technologies, and societies.
